# Directed evolution of the bacterial endo-β-1,4-glucanase from *Streptomyces* sp. G12 towards improved catalysts for lignocellulose conversion

**DOI:** 10.1186/s13568-018-0602-7

**Published:** 2018-05-05

**Authors:** Davide Agostino Cecchini, Olimpia Pepe, Anna Pennacchio, Massimo Fagnano, Vincenza Faraco

**Affiliations:** 1 Department of Chemical Sciences, University of Naples Federico II, Complesso Universitario Monte S. Angelo, Via Cintia, 4, 80126 Naples, Italy; 2 Department of Agricultural Sciences, University of Naples Federico II, Portici, Naples, Italy

**Keywords:** Directed evolution, High-throughput screening, Cellulase, *Arundo donax*, Saccharification

## Abstract

With the aim to develop biocatalysts for enhanced hydrolysis of (hemi)cellulose into monosaccharides, random diversity by directed evolution was introduced in the gene coding for the endo-β-1,4-glucanase from *Streptomyces* sp. G12 which had been recombinantly expressed in *Escherichia coli* and named rCelStrep. The main objectives were therefore to set up a complete strategy for creation and automated screening of rCelStrep evolved direct mutants and to apply it to generate and screen a library of 10,000 random mutants to select the most active variants. The diversity was introduced in the gene by error-prone polymerase chain reaction. A primary qualitative screening on solid plates containing carboxymethylcellulose as the substrate allowed selecting 2200 active clones that were then subjected to a secondary quantitative screening towards AZO-CMC for the selection of 76 improved variants that were cultured in flasks and characterized. Five rCelStrep mutants exhibiting the highest hydrolytic activities than the wild-type enzyme were further characterized and applied to the bioconversion of the pretreated *Arundo donax* lignocellulosic biomass. It is worth of noting that one of the five tested mutants exhibited a 30% improvement in bioconversion yields compared to the wild-type enzyme, despite the absence of the carbohydrate binding module domain in this variant. Homology models of the three-dimensional structures of the catalytic and binding modules of rCelStrep were obtained and localization of mutations on these models allowed us to speculate on the structure–function relationships of the mutants.

**Electronic supplementary material:**

The online version of this article (10.1186/s13568-018-0602-7) contains supplementary material, which is available to authorized users.

## Introduction

In recent decades, clean renewable resources, used as an alternative to fossil sources, have been becoming a main topic of research due to need to counteract the concerns regarding the shortage, the green-house gas emissions and global warming related to the latter ones (Liguori et al. 2013).

Lignocellulosic biomass represents the most abundant and sustainable one in nature, giving also the advantages to be widely spread geographically and also present in huge amounts of wastes and minimize the conflict of use for food versus fuel (Liguori and Faraco 2016).

Lignocellulosic biomasses can be hydrolysed in biorefineries processes requiring mixtures of enzymes that act on the complex carbohydrates matrix and consist of tailor-made cocktails of enzymes comprising different and complementary enzymatic activities, such as endoglucanase, β-glucosidase, cellobiohydrolase, xylanases, and arabinofuranosidase, with cellulases as main components (Van Dyk and Pletschke 2012).

However, these processes have to deal with two major bottlenecks: the high costs of currently adopted enzymes and the low yield in the hydrolysis due to the complex and recalcitrant nature of the biomass preventing the development of the biochemicals at competitive levels and impacting negatively on the total costs of biomass conversion process (Van Dyk and Pletschke 2012; Capolupo and Faraco 2016).

There is therefore an increasing demand for both enhanced bioconversion routes and novel cellulases with higher hydrolytic efficiencies and improved stability properties: great efforts have being hence employed for developing improved bioconversion conditions and reactor configurations (Liguori et al. 2016) and discovering novel enzymes and engineering the existing ones (Wang et al. 2012a). In this context, directed evolution is a process that more closely resembles natural protein evolution and is advantageous compared with rational design because it does not require a detailed knowledge of the target system (Tizei et al. 2016).

In this context, directed evolution of the α-l-arabinofuranosidase rPoAbf from *Pleurotus ostreatus* (Giacobbe et al. 2014) allowed us to develop an improved mutant with higher activity than the wild type enzyme. This variant was used in lignocellulosic biomasses (*Arundo donax*, corn cobs and brewer’s spent grains) conversion (Marcolongo et al. 2014), improving the sugars yields in comparison to the wild type enzyme.

In this work, this strategy was also applied to obtain mutants of the endo-β-1,4-glucanase activity (EC 3.2.1.4) CelStrep, that was previously isolated from the strain *Streptomyces* sp. G12 and recombinantly expressed in *Escherichia coli* and characterized (Amore et al. 2012). This recombinant cellulase rCelStrep was also tested in hydrolysis of the pretreated lignocellulosic biomass *Arundo donax* in substitution of commercial cellulases (Marcolongo et al. 2014) and in addition to commercial cellulases (Giacobbe et al. 2016), to evaluate it as a potential candidate for lignocellulose hydrolysis in biorefinery process but it was observed that its addition caused a decrease of the glucose and xylose yield. Therefore, the main objectives of this work were to set up a complete strategy for creation and automated screening of rCelStrep evolved direct mutants and to apply it to generate and screen a library of 10,000 random mutants, to select the variants with improved hydrolytic activities to be used for conversion of the biomass *Arundo donax.*

## Materials and methods

### Strains, media and chemicals

The *Escherichia coli* strain Top 10 (*F*-*mcrA D (mrr*-*hsdRMS*-*mcrBC) f80lacZDM15 DlacX74 deoR recA1 araD139 D (ara*-*leu) 7697 galU galK rpsL (StrR) endA1 nupG*) (Invitrogen) was used in all DNA manipulations and its grown was performed in Luria–Bertani (LB) medium (in g/L: 10 bactotryptone, 10 NaCl, 5 yeast extract), supplemented with 100 μg/mL of ampicillin for the selection of transformed clones.

The strain *E. coli* BL21 CodonPlus (DE3) RP (Novagen Ltd) was used as host for heterologous expression.

Gene Morph II Random Mutagenesis Kit used for error-prone (ep) PCR (polymerase chain reaction) mutagenesis was purchased from Agilent (La Jolla, California, USA). All restriction enzymes, T4 DNA ligase and their corresponding buffers were purchased from New England Biolabs (Beverly, MA, USA). DNA sequencing was performed by Eurogentec (Seraing, Belgium). AZO-CMC was obtained from Megazyme (Wicklow, Ireland). Accellerase 1500, Accellerase^®^ XY and Accellerase^®^BG were obtained from Genencor (Rochester, New York, USA).

### Construction of CelStrep error-prone PCR library

The template used for epPCR was the pET28a-celstrep construct harbouring the wild-type gene of CelStrep previously described (Amore et al. 2012). The following primers were used for the amplification: epCelstrepFW: CGGCCGCAAGCTTGGGGC; epCelstrepREV: GCGGCAGCCATATGCCTCG. Random mutagenesis was carried out using the Gene Morph II Random Mutagenesis Kit. Two different concentrations of template DNA were used, according to the manufacturer protocol, for the generation of medium mutation frequencies (MMF) and high mutation frequencies (HMF). Briefly, the PCR reactions mixture (50 µL) contained 500 ng of template DNA for MMF and 100 ng of DNA for HMF in the appropriate buffer, 1 mM of each NTP, and 1 IU of Polymerase. PCR reactions were carried out using the following program: one denaturation step at 95 °C for 2 min, 30 cycles composed of a denaturation step at 95 °C for 30 s, an hybridization step at 59 °C for 30 s, an elongation step at 72 °C for 30 s, and finally a 2 min step at 72 °C. 4 epPCR were carried out to obtain the amount of mutated CelStrep cDNA needed for construction of a library of 10,000 mutants. The obtained amplicons were purified using QIAquick Gel Extraction Kit (Qiagen, Hilden, Germany) digested with *Nde*I and *Hin*dIII and inserted into a similarly digested pET28a vector. The ligation mixture was used to transform chemically competent *E. coli* TOP10 cells and plated onto LB agar medium supplemented with ampicillin (100 µg/mL). After an overnight growth at 37 °C, colonies harbouring the epCelStrep encoding gene variants were collected by adding liquid LB medium containing ampicillin (100 μg/mL) to the plate surface and by suspending them with a scraper. The pulled cell suspension was centrifuged (5000×*g* for 10 min at 4 °C) and used to prepare purified plasmid DNA using a QIAprep Spin Miniprep Kit (Qiagen, Hilden, Germany). This DNA was used to transform chemically competent *E. coli* BL21(DE3) RP cells for heterologous expression of the random mutants’ library.

Transformed cells were plated on LB plates containing ampicillin (100 µg/mL) and incubated overnight at 37 °C to select the error-prone PCR CelStrep (epCelStrep) mutants.

### Screening of epCelStrep mutants library

The clones of *E. coli* transformed as above described were transferred from selective solid medium to 200 µL of liquid medium LB in 96-well plates with the robot colony picker QPIX 450 (Molecular Devices, LLC, California, USA). After overnight growth at 30 °C, the mutants were transferred on 22 × 22 cm Q-Tray bioassay plates containing LB-agar containing ampicillin (100 μg/mL) supplemented with 0.2% w/v carboxymethylcellulose (CMC) as substrate and 1 mM Isopropil-β-d-1-tiogalattopiranoside (IPTG) as inducer and the incubation was carried out overnight at 28 °C. Afterwards, to identify the colonies able to hydrolyse CMC the plates were stained with a solution of Congo red (1% w/v) for 15 min followed by 2 washing step of 15 min with a solution of 1 M sodium chloride. The clones showing activity halos were selected and transferred from 96-well plates to 96 deep-well microplates for the secondary screening, focused on the detection of variants with higher activity than the wild type enzyme in solution towards the chromogenic substrate AZO-CMC. After growth at 30 °C for 21 h, 50 µL of culture broth were transferred in 950 µL of liquid medium in 96 deep-well microplates by using the robot BioMek NXP (Beckman Coulture, California, USA) and the cultures were incubated at 37 °C for 4 h and then induced with IPTG for 3 h. After centrifugation at 5000×*g* for 30 min at 4 °C, samples of culture supernatant were subjected to analysis of endo-β-1,4-glucanase activity production towards the chromogenic substrate AZO-CMC.

Endo-β-1,4-glucanase activity produced in liquid culture was determined by transferring 50 µL of supernatant containing the secreted mutants or the wild-type enzyme into 96 deep well plates filled with 200 µL of AZO-CMC (1% w/v) diluted in 100 mM sodium acetate buffer pH 5 as the substrate. The deep well plates were incubated at 50 °C and after 15 min the assays were stopped by the addiction of 625 µL of precipitant solution, prepared following the supplier protocol, and by centrifuging the deep-well plates at 3700 rpm for 30 min at 4 °C. Finally 200 µL of the assays mixtures were transferred from the deep well plates into microplates and the absorbance of the soluble released products measured at 540 nm using a plate reader Multi Detection SystemGloMax^®^ Discover System (Promega, Wisconsin, USA).

### Expression of recombinant wild-type CelStrep and epCelStrep mutants in flasks

The wild-type CelStrep as well as the epCelStrep mutants were recombinantly expressed in 50 mL of LB medium supplemented with ampicillin (100 µg/mL) as previously described (Amore et al. 2012). Cells were grown with a rotary shaker at 37 °C until the OD600 reached a value between 0.8 and 1.0. Then the cultures were induced with 1 mM IPTG and the expression was performed at 28 °C for 18 h. Liquid cultures were centrifuged at 5000×*g* for 30 min at 4 °C to remove cells and the supernatants containing cellulase activity were then used for enzymatic assays, enzymatic properties determination and biomass hydrolysis.

### AZO-CMC assay

Endo-1,4-β-glucanase activity produced in liquid culture was assayed by using AZO-CMC (Megazyme, Ireland) as substrate, following supplier instructions and determined by referring to a standard curve. The absorbance of the reaction solutions was measured at 590 nm. One unit of enzyme is defined as amount of enzyme catalyzing the release of 1 μmol of glucose equivalent per min. The reported results correspond to mean values of three independent experiments, each one performed in three replicates.

### Optimum pH, temperature and thermoresistance

The supernatant of liquid cultures of *E. coli* clones expressing wild-type rCelStrep and the selected mutants was concentrated by using the stirred ultrafiltration Amicon^®^ system (Millipore Corporation, Bedford, MA, USA) with a 3 kDa polyethersulfone membrane. The concentrated enzymatic extract was used to determine the optimum temperature and pH and thermo-resistance of the cellulase activity. The pH dependence of enzyme activity was determined between pH 4.0 and 6.0 by dissolving AZO-CMC in the appropriate sodium acetate buffer at 50 °C. The temperature dependence of enzyme activity was determined between 40 and 60 °C in sodium acetate buffer pH 6.

Thermostability of the enzymes was determined by incubating the enzymes in 100 mM sodium acetate buffer pH 6 at 40, 50 or 60 °C. At various times, aliquots of enzymes were removed and assayed for residual activity using AZO-CMC as the substrate. The reported results correspond to mean values of three independent experiments, each one performed in three replicates.

### Enzymatic hydrolysis

*Arundo donax* pretreated according to Garbero et al. (2010) and De Bari et al. (2013) was utilized as substrates as slurry without any downstream process in biotransformation experiments carried out in capped tubes, on the rotary shaker ThermoMixer C (Eppendorf, Milan, Italy) at 6000 rpm.

The hydrolysis of pretreated lignocellulosic materials with the enzymatic cocktails was carried out at a concentration of pretreated biomass of 10% (w/v) in a total volume of 2.5 mL of 100 mM sodium acetate buffer pH 6.0. The components of the enzymatic commercial cocktail adopted as benchmark were Accelerase 1500, Accelerase BG and Accelerase XY provided by Genencor and were prepared at the amounts expressed as units per gram of pretreated biomass: 5.4, 145 and 4000, respectively. Saccharification performances of the tested enzymatic extracts were evaluated replacing Accelerase 1500 by each of the tested extracts (wild-type CelStrep or epCelStrep mutants) using the same amounts of units per gram, namely 5.4 units/g of pretreated biomass. The reactions were incubated at 600 rpm and 50 °C. The samples were withdrawn at different time intervals (0, 24, 48 and 72 h), cooled on ice and centrifuged at 16,500×*g* for 30 min at 4 °C. The supernatants were analysed to quantify the amount of sugars released by high-performance liquid chromatography (HPLC; Dionex, Sunnyvale, California, USA) using the protocol described in Ventorino et al. (2016).

### Homology modelling

The three dimensional structures of the two domains of the CelStrep enzyme were determined by submitting the translated celstrep gene to SWISS-MODEL (http://expasy.org/). The crystal structure of the CelB2 1,4-β-glucanase from *Streptomyces lividans* PDB ID: 2NLR (sequence identity 84%), was used as a template for the molecular model of the CelStrep catalytic domain. To map the substrate-binding subsites the structures of CelB2 from *S. lividans* and cellulose 12A from *Thermotoga maritima* PDB ID: 3AMP (sequence identity 22%), in complex with cellotriose and cellobiose, respectively, were superimposed on the top of the structure of CelStrep (Sulzenbacher et al. 1997, 1999). The crystal structure of Cex CMB2 from *Cellulomonas fimi* PDB ID: 1EXG (sequence identity 54%), was used as a template for the molecular model of CelStrep CBM (Xu et al. 1995). Structure visualisation and analysis were achieved using PyMOL (DeLano 2002).

## Results

### Construction and screening of rCelStrep error-prone PCR library

In the present study, an automated workstation including the robot colony picker QPIX 450 (Molecular Devices, LLC, CA, USA) and the robot BioMek NXP (Beckman Coulter, CA, USA) was adopted for the library screening and an ad hoc strategy of automated HTS for variants endowed with higher cellulolytic activity developed (Additional file 1: Fig. S1). In particular, the robot colony picker QPIX 450 was used to transfer the obtained clones of *E. coli* from selective solid medium to 96-well plates containing LB medium (Additional file 1: Fig. S1a, picking). After overnight growth, the mutants were transferred on Q-Tray bioassay plates containing LB solid medium (Additional file 1: Fig. S1b, gridding) and activity was detected after Congo Red staining. A total of 2220 clones, corresponding to around 20% of the library, resulted to be active and able to degrade CMC, forming halo around active colonies after Congo Red staining.

The clones showing activity halos were selected and transferred from 96-well plates to 96 deep-well microplates (Additional file 1: Fig. S1c, re-arraying) for the secondary screening, focused on the detection of variants with higher activity than the wild type enzyme in solution towards the chromogenic substrate AZO-CMC. The β-1,4-glucanase activity production was detected after centrifugation (Additional file 1: Fig. S1e) using substrate AZO-CMC (Additional file 1: Fig. S1f). Among the assayed clones, 76 mutants showing a six- to sevenfold higher activity than the wild-type enzyme were selected and further screened in flasks, leading to the selection of the best five variants exhibiting up to two- to threefold higher activity compared to the wild-type enzyme (Table 1).

**Table 1:** Activity value of wild type rCelStrep and mutants, nucleotide and aminoacid substitutions in rCelStrep variants

	mU/mL	mU/OD600	Nucleotide substitution	Aminoacid substitution
CelStrep wild type	70.4 ± 10.8	984.6 ± 52.3	–	–
epCelStrep_1 (epCS_1)	103.8 ± 0.3	1222.2 ± 94.2	G898T, G1031A	G263C; R307H
epCelStrep_2 (epCS_2)	88.6 ± 8.5	1711.9 ± 456.4	G49A, G545A, T732A, G858A, C986A, C1131A	G145D; N207K
epCelStrep_3 (epCS_3)	112.6 ± 6.7	3295.2 ± 202.1	C411T, T687C, C794G, G857A, G954T, G1060A, C1130T	P228R
epCelStrep_4 (epCS_4)	89.1 ± 6.6	1095.1 ± 44.9	C311A, T537G, G808A, A828G, T836A, T1101A	T67N; D142E; S218N; V242D; D330E
epCelStrep_5 (epCS_5)	102.9 ± 0.2	2073.3 ± 689.1	C363T, C581T, G863A, T887A	T157I; G251D; V259D

AZO-CMCase activity levels of the CelStrep wild type and of the five evolved enzymes expressed as mU/mL and normalized to OD_600nm_. The enzymes were assessed for the endoglucanase activity in liquid medium in the presence of 1% CMC at 50 °C. Experiments were performed in triplicate. Nucleotide and aminoacid substitutions in the selected rCelStrep variants

Scaling-up the growth of the wild type and evolved variants to 50 mL in flask resulted in a lower cellulase activity production. This result is due to changes of growth conditions in comparison with micro-scale (Burke 2008).

The best five mutants in terms of higher activity were sequenced and the corresponding mutations of the selected clones are reported in Table 1.

### Enzymatic properties of the five rCelStrep mutants: optimal pH and temperature and thermostability

**Fig. 1:**
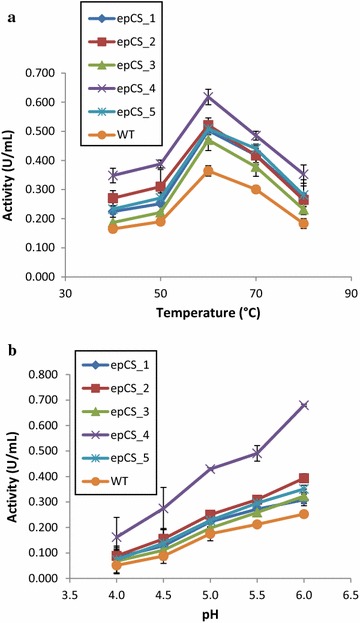
Temperature (**a**) and pH (**b**) dependence of endo-glucanase activity for wild-type CelStrep and mutants. For the optimal temperature endo-glucanase activity was measured at pH 6 and temperatures ranging from 40 to 80 °C, using AZO-CMC as substrate. For the optimal pH determination, the endo-glucanase activity was measured in Na acetate buffer at pH ranging from 4 to 6, using AZO-CMC as substrate

To establish the optimal conditions of pH and temperature for the application of the five selected mutants to lignocellulose hydrolysis in bioconversions, the enzymatic properties were investigated on the culture supernatants using AZO-CMC as substrate. Figure 1a, b show that the activity profiles of the five mutants at the investigated values of pH and temperature were similar to that of the wild-type enzyme, with the highest activity at pH 6 and 60 °C, in the investigated conditions.

As enzymatic bioconversions take some days, the thermo-resistance of the enzymes is an important factor for the success of the process. Therefore, we determined and compared the thermostability of the five mutants to the wild-type enzyme. All the mutants as well as the wild-type enzyme were stable at 40 and 50 °C, showing a retention of around 80% of activity after 72 h (data not shown). Similar results were reported for the purified wild-type enzyme in a previous paper (Amore et al. 2012). The thermostability of the five mutants at 60 °C was instead different, with epCelStrep_1 and epCelStrep_3 showing an improvement of stability, after 72 h, and retaining slightly higher activity (34%) compared to epCelStrep_2 (10%), epCelStrep_4 (10%) and the wild-type enzyme (17%).

### Evaluation of mutations in selected variants

**Fig. 2:**
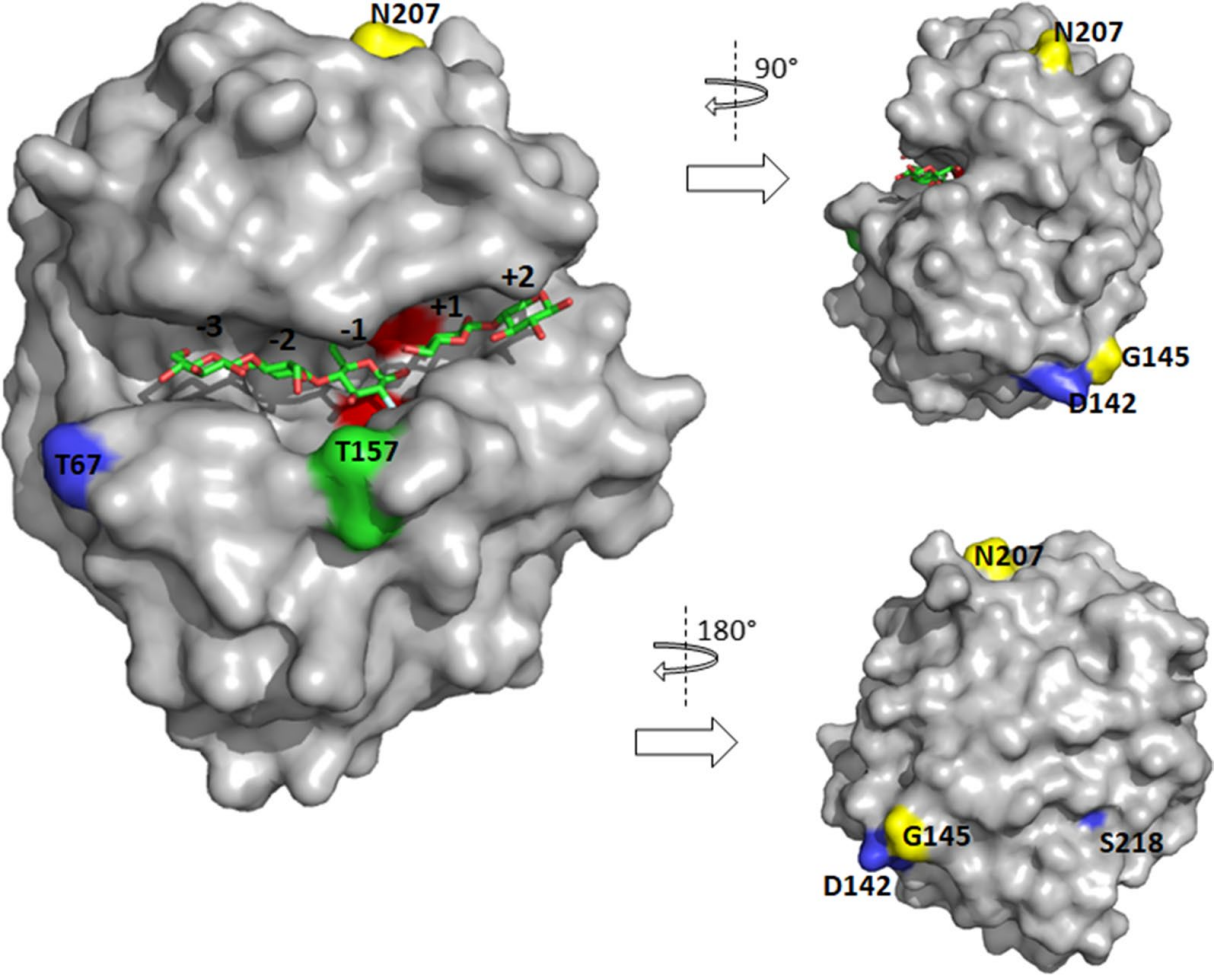
Homology model of the catalytic module of CelStrep. Surface view of the catalytic cleft of CelStrep with cellotriose modelled in the − 1, − 2 and − 3 subsites and cellobiose modelled in the + 1 and + 2 subsites. The two catalytic glutamate residues, E120 and E203, are coloured in red. The substituted labelled amino-acids are coloured according to the epCelStrep variant: epCS_2 (yellow), epCS_4 (blue), epCS_5 (green)

**Fig. 3:**
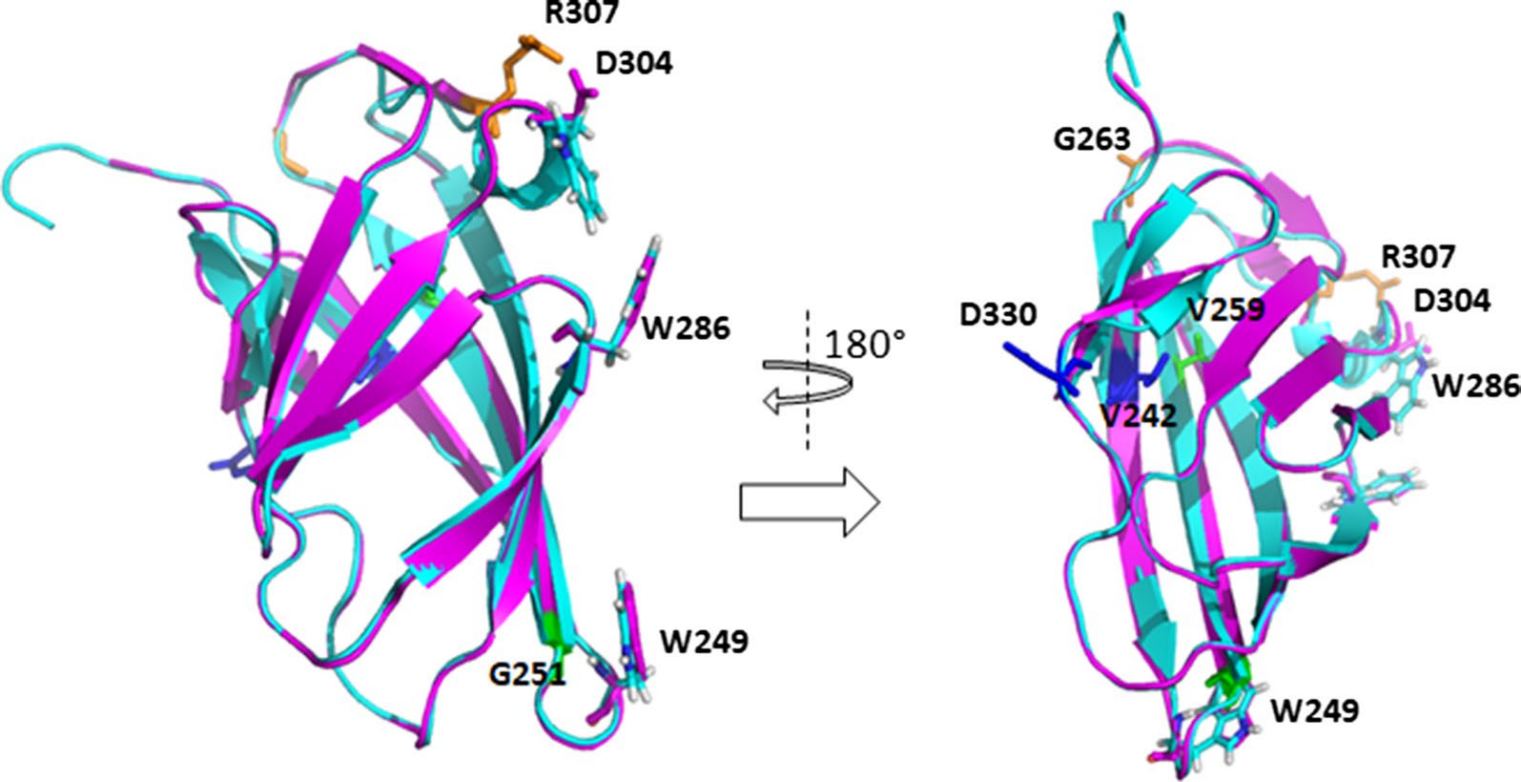
Homology model of CBM structure of CelStrep overlaid to CBM of Cex from *Cellulomonas fimi*. Ribbon view of the structural overlay of the CBM of CelStrep (magenta) with the CBM of *C. fini* (cian). The three substrate binding tryptophan residues of *C. fini* are coloured in cian and the corresponding residues, W249, W286 and D304 of *Streptomyces* sp. GH12 in magenta. The substituted labelled amino-acids are coloured according to the epCelStrep variant: epCS_1 (pink), epCS_4 (blue), epCS_5 (green)

Homology models of the three-dimensional structures of the catalytic (Fig. 2) and binding (Fig. 3) modules of rCelStrep from *Streptomyces* sp. G12 were obtained. With a 37,000 Da molecular mass, this enzyme is composed of a signal peptide for secretion, of 37 aminoacids, a catalytic module belonging to the GH 12 family, of 222 aminoacids, bound through a small linker region to a carbohydrate binding module (CBM) belonging to the family CMB2 (Henrissat and Bairoch 1996) of 107 aminoacids.

Examination of the model structure revealed that the aminoacidic substitutions were introduced not only in the catalytic module but also in the CMB as well as the linker region binding them. More in particular: in epCelStrep_1, two substitutions (G263C and R30H) were introduced in the CBM; in epCelStrep_2 two substitutions (G145D and N207K) were introduced in the catalytic module; in epCelStrep 3, the only substitution (P228R) was introduced in the linker region; in epCelStrep_4, five substitutions were introduced of which three (T67N, D142E and S218N) in the catalytic module and two (V242D and D330E) in the CBM; in epCelStrep_5, one substitution (T157I) was introduced in the catalytic module and two (G251D and V259D) in the CBM.

### Saccharification of pretreated biomass of *A. donax* by rCelStrep mutants and wild-type enzyme

**Fig. 4:**
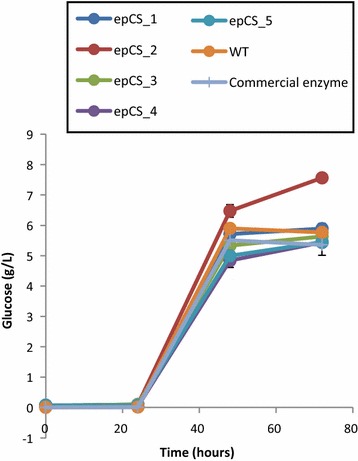
Enzymatic hydrolysis of pretreated *A. donax* biomass. Hydrolysis reactions were performed in Na acetate pH6 and 50 °C using cocktails prepare with commercial enzymes (xylanase and β-glucosidase), in combination with CelStrep WT and epCS mutants

To evaluate the five rCelStrep selected mutants as well as the wild-type enzyme in hydrolysis of *A. donax* biomass, they were used to replace the cellulase Accelerase^®^ 1500 in a cocktail containing the commercial xylanase Accelerase XY and β-glucosidase Accellerase^®^ BG and the amount of glucose released was determined by HPAEC-PAD and it is reported in Fig. 4.

The enzymes from epCelStrep_wt, epCelStrep_1, epCelStrep_2 and epCelStrep 3 led better results than those obtained with the commercial enzymes. On the other hand the enzymes from epCelStrep_4 and epCelStrep_5 give results similar to those obtained in the presence of commercial enzymes.

## Discussion

To improve the enzymatic activity of the β-1,4-endo-glucanase from the strain *Streptomyces* sp. G12 recombinantly expressed in *E. coli* BL21(DE3) and named rCelStrep (Amore et al. 2012), this enzyme was subjected to random biodiversity generation applying an error-prone PCR (epPCR) mutagenesis approach on the corresponding gene.

From preliminary studies, it was observed that the introduction of few mutations (1–3) per kb has little effect on the enzyme activity, with the majority of the variants still active (data not shown). On the other hand, it has been reported that the introduction of very higher rates of mutations has dramatic consequences on the enzyme activity and/or stability (Wang et al. 2012b; Song et al. 2012). Therefore, two different PCR conditions to yield medium mutation frequency (MMF: 4–9 mut/kb) and high mutation frequency (HMF: 9–16 mut/kb) were adopted in this work, and a library of 5000 MMF and 5000 HMF mutants was generated.

Although directed evolution has been shown to be a potent technology for the improvement of enzymes that are potentially important for industrial applications, there are several limitations that need to be solved, mainly related to the lack of rapid and generalized high-throughput screening (HTS) systems. In the present study, an ad hoc strategy of automated HTS for variants endowed with higher cellulolytic activity was developed. The main peculiarity of this strategy was the development and use of a method for high-throughput screening in liquid based on the substrate AZO-CMC instead of other more laborious methods, so far adopted, as for example the 3,5-dinitrosalicylic acid methods that are based on the analysis of the reducing sugars produced by the hydrolysis of cellulose (Percival-Zhang et al. 2006). Applying this screening strategy to the library of 10,000 rCelStrep variants, the best five mutants in terms of higher activity were selected for further analyses.

From sequence analysis of these mutants, it was observed that there were not amino acid substitutions at positions 120 and 203 corresponding to the positions of the potential catalytic glutamates, nucleophile and acid/base, respectively, in the sequence of wild type rCelStrep. Moreover, as a result of the aminoacidic sequence analysis, it is worth of nothing that the variants epCelStrep_2 and epCelStrep_3 resulted to be truncated proteins. In particular the two mutants miss the CBM, due to the introduction of a stop codon between the catalytic module and the CBM at the position 249. It was already reported that the endo-glucanase activity on soluble substrates, like AZO-CMC, is not affected by the removal of the CBM, whilst the partial or complete loss in catalytic activity could be observed on insoluble substrates (Kleine and Liebl 2006; Waeonukul et al. 2009). Moreover, the analysis showed that epCelStrep_3 and epCelStrep_2 have also a single (P228R) and a double substitution (G145D/N207K), respectively. The analysis of the other three variants revealed the introduction of two G263C/R307H (epCelStrep_1), three T157I/G251D/V259D (epCelStrep_5) and five T67N/D142E/S218N/V242D/D330E (epCelStrep_4) substitutions located in the catalytic domain and in the CBM.

Examination of location of the aminoacidic substitutions on the 3D homology models led to the following considerations. The epCelStrep_1 mutant revealed that the two substitutions introduced, G263C and R307H, are localized in the CBM. The CBMs belonging to family 2 are dividend in two classes, 2a and 2b, according to the two substrate they bound, cellulose or xylan, respectively. It has been shown that three tryptophan residues, W17, W54 and W72 in the three dimensional structure of the type 2a CBM from *Cellulomonas fimi* cellulase Cex (CBMCex) play an important role in binding both soluble and insoluble cellulose (Nagy et al. 1998). These tryptophan are surface exposed and oriented to form an flat aromatic strip ideally placed to interact with the flat surface of cellulose. As shown in the structure alignment in Fig. 3, of the three residues present in CBMCex, W17, W54 and W72, only the first two, corresponding to the equivalent W249 and W286, are present in the CelStrep CBM while the third one is replaced by an aspartic acid residue (D304). But one of the two substitutions identified in the epCelStrep_1 mutant, R307H, is only three residues far from D304. We can speculate that the introduction of an histidine with its aromatic ring very close to the D304 position could have extended the aromatic strip formed by W249 and W286, those increasing the affinity of CelStrep for the substrate.

The two substitutions introduced in the epCelStrep_2 mutant, G145D and N207 K, are located instead on the surface of the GH12 catalytic module. In particular N207 K is located only three aminoacid far from the general acid/base catalyst E203, involved in the protonation of the substrate. We can speculate that this substitution could have introduced structural modification in the active site responsible of the improved biomass degrading performances showed by this mutant.

Surprisingly the only mutation identified in the epCelStrep_3 mutant, P228R, is located in the linker region normally connecting the catalytic domain to CBM module, the latter one lacking in this mutant. However, improvement in hydrolytic activity caused by a proline substitution in the linker region of multi-modular proteins, have been previously described (Couturier et al. 2013).

The epCelStrep_4 mutant presented the highest number of the substitutions, with three substitutions, T67N, D142E and S218N, localized in the catalytic module and two, V242D and D330E, in the CBM. As Fig. 2 shows, among the three mutations introduced in the catalytic module, T67N is close to the substrate subsite − 3, those in a position to influence the binding of the substrate and as a consequence the performance of the enzyme. The fact that the third mutation introduced, D142E, has been found near the G145D substitution, identified in the epCelStrep_2 mutant, could suggest a possible hot spot region for enzyme improvement to be further explored. The last two substitutions identified, V242D and D330E, are located in the CBM and far from the binding site.

Among the three substitutions present in the variant epCelStrep_5, T157I is part of a loop which has been shown having various roles in catalysis. In particular it has been shown that removing the loop sequence (STTQA) containing this residue compromises the catalytic efficiency suggesting an important role in substrate hydrolysis and product release (Yang et al. 2017). The other two substitutions, G251D and V259D, are located in the CBM. In particular, G251D is close to G252, a residue strictly conserved in CBM2a that sits partially under the aromatic ring of W249. It has been shown to be important for the correct orientation in the *C. fimi* CBMCex of the tryptophan W17, equivalent to the CelStrep W249. Its substitution with and an arginine causes the rotation of W17 by approximately 90°, converting the CBM from a cellulose to a xylan binder (Simpson et al. 2000). Nevertheless, the fact that the mutant was active against AZO-CMC, suggest that the substitution G251D, at least in combination with V259D, have a different impact on substrate specificity.

To evaluate the applicability of the five rCelStrep mutants in lignocellulose bioconversions, these variants were tested in the hydrolysis of pretreated biomass of the giant reed *Arundo donax* and their performances were compared to those of the wild-type enzyme.

It is worth of noting that the glucose amount obtained with the wild-type rCelStrep was slightly higher than that obtained with the commercial enzymes, differently from what reported in our previous work (Marcolongo et al. 2014), that can be ascribed to the different operative conditions hereby applied. Moreover, among the tested enzyme mixtures, the one prepared with the mutant epCelStrep_2 led to improvements in sugar conversions. Indeed, by using this variant in the enzyme mix the amount of glucose released after 72 h was 30% higher compared to the mix prepared with the wild-type enzyme, despite the absence of the CBM domain in this variant. This behaviour can be considered coherent with the higher hydrolytic activity of this mutant towards AZO-CMC, considering that the absence of CBM normally does not affect hydrolysis of soluble substrates, but also with the high concentration of pretreated biomass adopted in bioconversions (10%), in agreement with previous observation that reduced amount of water counterbalances the need for CBMs in hydrolysis of insoluble substrates (Várnai et al. 2013). However, the yields in sugar hydrolysis obtained with the mixes prepared with the other mutants, even if still endowed with higher activity towards AZO-CMC than the wild-type enzyme, were instead similar to those obtained with the mix prepared with the latter enzyme. These results could be explained by hypothesizing an improvement of the synergism between the mutant epCelStrep_2 and the other two enzymes (xylanase and β-glucosidase) used for the preparation of the cocktail, due to the mutations introduced, in comparison to the other tested mutants.

As a matter of fact, it has been reported that in the formulation of cocktails, by using enzymes from different sources but active on the same substrate different levels of synergism can be afforded (Woodward 1991; Morrison 2014).

In conclusion, this work allowed introducing random diversity in the gene coding for the cellulase from *Streptomyces* sp. G12 expressed in *E. coli* rCelStrep by epPCR, generating a library of 10,000 variants that were screened by an ad hoc strategy of automated HTS for variants with higher cellulolytic activity. This allowed selecting five variants exhibiting higher activity towards AZO-CMC that were also tested in hydrolysis of pre-treated biomass *Arundo donax*. It is worth of noting that one of the five tested mutants exhibited a 30% improvement in bioconversion yields compared to the wild-type enzyme, despite the absence of the CBM in this variant.

## Additional file

**Additional file 1: Fig. S1.** Schematic representation of the high-throughput screening strategy developed to analyze *E. coli* mutants expressing CelStrep activity.

## Abbreviations

CMC: carboxymethylcellulose
LB: Luria–Bertani
*E. coli*: *Escherichia coli*
epPCR: error-prone polymerase chain reaction
MMF: medium mutation frequencies
HMF: high mutation frequencies
IPTG: isopropil-β-d-1-tiogalattopiranoside
*S. lividans*: *Streptomyces lividans*
*T. maritime*: *Thermotoga maritime*
*C. fimi*: *Cellulomonas fimi*
CBM: carbohydrate binding module
HTS: high-throughput screening
CBMCex: CBM from *Cellulomonas fimi* cellulase Cex
