# Supplementary material for: Directed evolution of the bacterial endo-β-1,4-glucanase from Streptomyces sp. G12 towards improved catalysts for lignocellulose conversion
Source: AMB Express. 2018 May 5;8:74. doi: 10.1186/s13568-018-0602-7 (PMC5935602; doi:10.1186/s13568-018-0602-7)

- Journal name: AMB Express

- Manuscript Title: Directed evolution of the bacterial endo-β-1,4-glucanase from *Streptomyces* sp. G12 towards improved catalysts for lignocellulose conversion

- The names of the authors: Davide Agostino Cecchini^1^, Olimpia Pepe^2^, Anna Pennacchio^1^, Massimo Fagnano^2^ and Vincenza Faraco^1^

- The affiliations and addresses of the authors: ^1^Department of Chemical Sciences, University of Naples Federico II, Complesso Universitario Monte S. Angelo, via Cintia, 4 80126 Naples, Italy ^2^Department of Agricultural Sciences, University of Naples Federico II, Portici (Naples), Italy

- E-mail address, telephone and fax numbers of the corresponding author: email address: vfaraco@unina.it; telephone number: +39 081674315; fax number: +39 081674313

**Growth by 21h incubation at 30°C (200 rpm); 1:20 dilution and growth by 4h incubation at 37°C (200 rpm) followed by 3h induction with 1 mM IPTG**

**Figure S1**. Schematic representation of the high-throughput screening strategy developed to analyze *E. coli* mutants expressing CelStrep activity


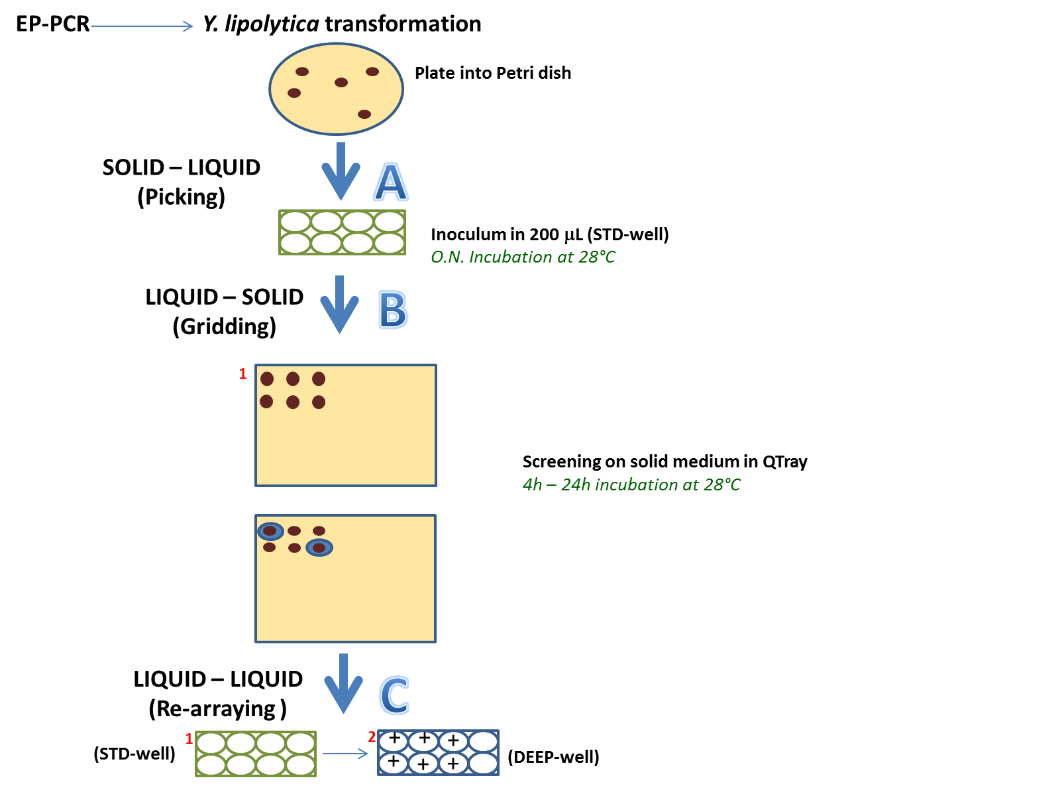


*E. coli* BL21 CodonPlus (DE3) RP transformation

**Screening on LB + Agarose 1%**

**towards 0.2 % CMC in the presence of 1 mM IPTG**

**16-18h incubation at 30°C followed by Congo Red staining**

**Overnight growth at 30°C (200 rpm)**


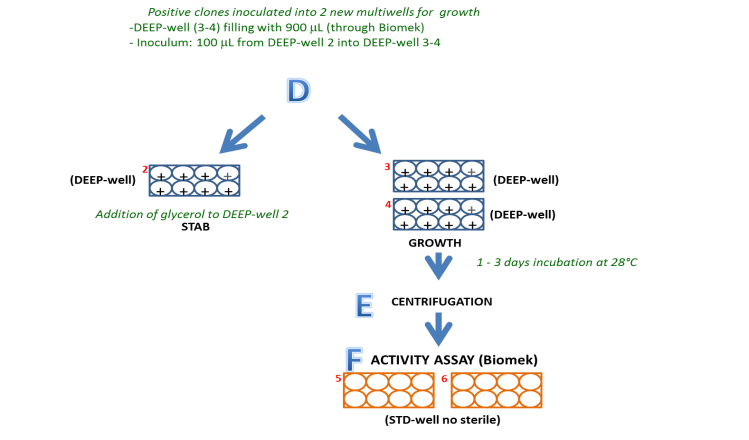

Supplement: Supplementary file 1 — Additional file 1: Fig. S1. Schematic representation of the high-throughput screening strategy developed to analyze E. coli mutants expressing CelStrep activity. [file 13568_2018_602_MOESM1_ESM.docx]
